# The effect of resistance band exercises on motor skills in female gymnasts

**DOI:** 10.1186/s13102-026-01852-0

**Published:** 2026-07-06

**Authors:** Gül Halime Çelik, Sebiha Gölünük Başpınar

**Affiliations:** 1 Afyon TED College, Afyonkarahisar, Türkiye; 2 Department of Physical Education and Sports, Faculty of Sport Sciences, Afyon Kocatepe University, Afyonkarahisar, Türkiye

**Keywords:** Resistance Exercises, Theraband, Balance, Jumping, Strength, Gymnastics

## Abstract

**Background:**

In gymnastics, where repetitive loading, sudden movements, and high-effort techniques are common, the use of adjustable elastic bands, tailored to individual differences, can safely support children’s motor development. Therefore, in our study, we examined the effects of elastic resistance band (ERB) exercises on motor children in gymnasts aged 8–12 years.

**Methods:**

The study group consisted of girl children who had been actively practicing gymnastics for 3 years. A quantitative pre- and post-test control group experimental design, a type of experimental study, was used in the study. Participants were divided into 2 groups (the Elastic Resistance Band Group (ERBG) (n:11, year 10 ± 0.8) and the Control Group (CG) (n:10, year 10 ± 0.45). Both groups continued their routine gymnastics training three days a week for six weeks, but the ERBG underwent a 45-minute Resistance Band Exercise Program in addition to their gymnastics training. Physical measurements (height, weight, Body Mass Index) and motor tests (muscle power, flexibility, strength, balance, 20 m sprint) were administered to the groups as Pretests and Posttests.

**Results:**

The results showed no statistically significant differences between the ERBG and CG Pretest values. Significant differences were detected in the ERBG in pre- and Posttest flexibility, right and left foot balance, muscle power, and back strength values. In the CG group, there were no differences in any parameters except for left foot balance values.

**Conclusions:**

ERBG were found to be effective in improving motor skills in gymnastics. We recommend adding ERB exercises to gymnastics training to contribute to the development of motor skills.

**Supplementary Information:**

The online version contains supplementary material available at https://doi.org/10.1186/s13102-026-01852-0.

## Introduction

Gymnastics involves various movements and requires motor skills, suitable for people of all ages [1]. Basic gymnastics and rhythmic gymnastics training have positive effects on the motor skills of developing children [2]. Previous studies reported that boys are better at running, jumping, strength, and coordination, while girls are better at flexibility, agility, and balance [3, 4]. A gymnast’s success depends on the level of motor abilities, particularly strength. Strength occupies the highest position in the hierarchy of motor skills [5, 6]. The persistent decline in muscular fitness measurements among today’s childrens indicates a need for resistance training, not only for athletes but also for non-athletic youth [7]. When provided with appropriate guidance and training, resistance training offers numerous health and fitness benefits for children and adolescents, improving muscle strength and endurance [8, 9], performance characteristics such as vertical and horizontal jumps, and sprint speed [10, 11], bone mineral density, and reducing sports injuries [12]. It can even be used as a rehabilitation or preventative method for children [13].

There are several methods to improve strength, which include using one’s own body weight as well as various machines and equipment [14]. One of these pieces of equipment is elastic resistance bands (Therabands). It was reported that resistance exercise applications using different training methods (e.g., plyometric training, machine-based exercises, and resistance band exercises) have the potential to improve health-related outcomes (such as body composition and psychological well-being) and performance-related parameters (such as muscle strength and strength gains) in children [15]. Therabands can contribute not only to strength development but also to the development of other motor skills because they offer versatile movement and allow the user to feel the applied movement from all angles. Their usability across all age groups is another advantage [16]. For this reason, exercises performed with resistance equipment like Therabands can be more challenging and can also facilitate certain movements [17]. Coaches prefer these bands because of their low cost [14, 18], versatility, ease of use [18], and portability [16]. Theraband Exercises are used as part of training in many sports, including javelin throwing, mountain games, shot put, and discus throwing, as well as American football, wrestling, athletics, rowing, basketball, soccer, and rugby [19]. It was speculated that elastic band training also improves jumping performance and other volleyball-related parameters (anteroposterior balance, dynamic strength) in male volleyball players, and that it should be added to volleyball training [20].

Previous studies conducted on the use of elastic bands exist in various disciplines [21, 22] and in older age groups [23–25]. Research in gymnastics [26, 27] is limited, and studies conducted on resistance bands in child age groups remain unclear. Based on these research findings, it is suggested that elastic band exercises may be preferred in gymnastics training. The research findings can be used by sports scientists, sports experts, and coaches. This study aimed to investigate the effects of elastic band exercise applications on the balance, endurance, stamina, and strength values of gymnasts.

1. Are there a significant difference in post-test motor skill values between ERBG and CG who received gymnastics training supplemented with ERB exercises?

## Methods

### Research model

This study used a pre-test–post-test control group experimental research design, a quantitative research method. The research group consisted of gymnastics team athletes who regularly trained and had not taken long-term breaks from training, participating in provincial and regional competitions.

### Participants

The study group consisted of healthy girl children aged 8–12 who had been actively participating in gymnastics at the Gymnastics club for at least 3 years. Participants were included in the study using a purposive sampling method.

**Fig. 1:**
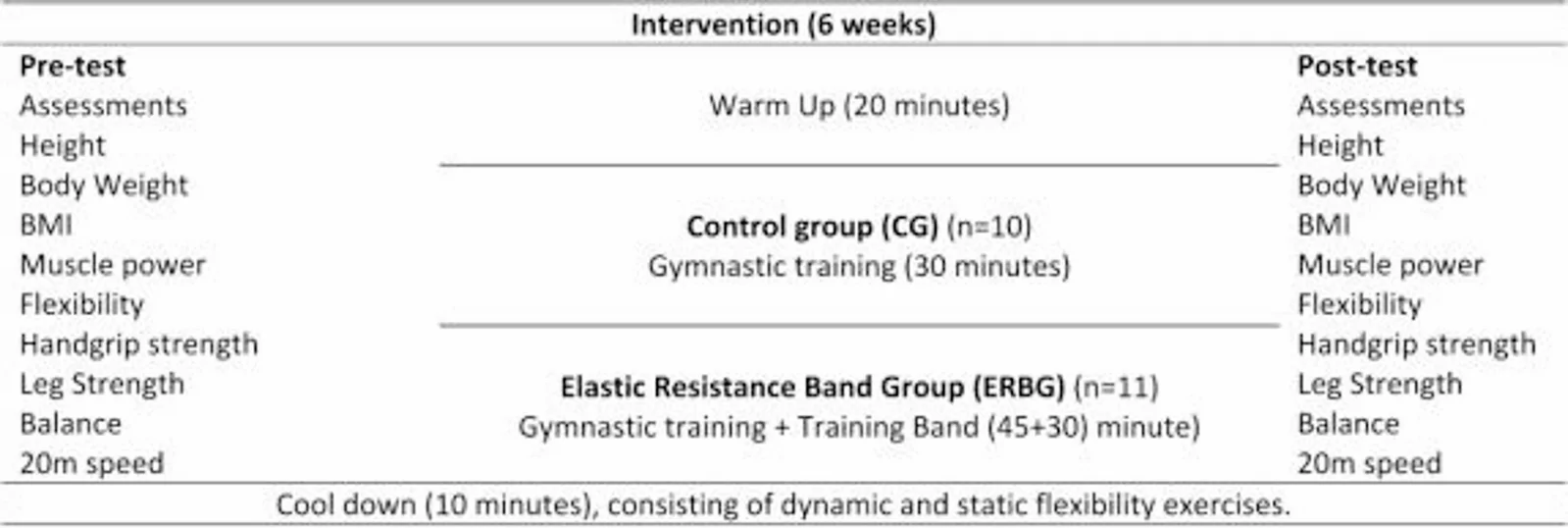
Study program flowchart

Inclusion criteria were being female gymnasts with no medical history, having been actively practicing and competing for at least 3 years. Gymnasts with any medical history or who had taken a break from active gymnastics within the last 3 years were excluded from the study. Participants were randomly assigned to the study. Participants were grouped according to the inclusion criteria determined before the study. To ensure similar baseline characteristics (performance levels), pre-test measurements were considered, and participants were then assigned to experimental and control groups. Power analysis regarding sample size was calculated using G*Power 3.1 software. The significance level was set at α = 0.05 and the sustained power (1 − β) = 0.80, with groups of n1 = 10 and n2 = 11, respectively. The programming results showed that the minimum effect size detectable with these dimensions is d ≈ 1.16. A total of 21 children were included in the study, divided into 2 groups: ERBG (*n* = 11) and CG (*n* = 10). Both groups continued their routine training for 60 min (warm up, gymnastic training, and cool down) a day, 3 days a week for 6 weeks (Fig. 1). However, in addition to training, the ERBG group also underwent 45 min of green Theraband exercises [28]. In the study, a flowchart is presented, inspired by Valdés-Badilla et al. [29].

### Ethical Approval

The study was conducted in line with the Declaration of Helsinki Principles, which govern research involving human participants. Ethical approval (2019/28) was obtained from the Afyon Kocatepe University Health Sciences Scientific Research and Publication Ethics Committee, along with institutional and parental consent. Before the study, parental and institutional consent were obtained, and a voluntary consent form was signed.

### Data collection tools

The age, height, body weight, and BMI (kg/m²) of the ERBG and CG were recorded. The groups then underwent a sit-and-reach flexibility test (baseline), static balance (flamingo), muscle power (vertical jump, fit jump), back and leg strength test (Takei Physical Fitness Test), hand grip strength test (Takei Hand Grip Dynamometer), and a 20 m sprint test before and after exercise. Participants were first shown all tests once and asked to try them out. All measurements were taken in 3 repetitions, and the best times were recorded.

#### Height and body weight (kg)

The athlete’s height (cm) was measured with a steel tape measure, barefoot, with heels together, knees straight and upright, and body in an upright position, with an accuracy of 1 mm. Body weight (kg) measurements were taken using bioelectrical impedance.

#### BMI

Calculated as Body weight kg/Height^2^ [30].

#### Muscle power

Muscle power is measured by the vertical jump test, which is based on the time spent in the air. In this test, participants are asked to jump as high as possible without pausing, keeping their hands on their hips and their bodies upright [31].

#### Flexibility (baseline sit and reach)

Participants are asked to reach as far as they can by bending their bodies forward without bending their knees, then slowly push the ruler forward and remain motionless at the furthest point for at least 2 s. Measurements were recorded in centimeters [32].

#### Handgrip strength

Grip strength (kg) was measured using a Takei digital (Model TKK 5401 Japanese) handgrip dynamometer. The dynamometer was adjusted as needed according to the participant’s hand size. Participants were instructed to grip the dynamometer with maximum force using both hands, keeping their wrists in a neutral position and their elbows away from their bodies. Children were asked to press the dynamometer for 3–5 s. Three tests were performed for each hand, and an average score was taken [33].

#### Leg and back strength

The participants were asked to grip the Takei brand digital back and leg dynamometer bar with their hands, with their knees bent, back straight, arms extended, and torso slightly leaning forward, and pull it vertically upwards to the maximum extent [34].

#### Balance

While balancing on the preferred leg, the free leg was lowered at the knee and kept close to hip level [35]. The number of falls was recorded over 60 s. If more than 15 falls occurred in the first 30 s, the test was terminated. A flamingo balance board measuring 50 cm long, 4 cm high, and 3 cm wide was used [36].

#### 20 m sprint

The participant places the tip of one foot on the starting line. Starting from a 0 m area, they ran a distance of 20 m at maximum speed with a high start. The time was recorded in seconds using a stopwatch [37].

### Gymnastics program

Gymnasts were competitive athletes who had been participating in the gymnastics club of a private school for at least 3 years without interruption and competing in competitions. Gymnastics practices were performed in the well-equipped gymnastics hall in the school. Floor gymnastics movements were performed during routine and practice. Training sessions lasted 60 min, with the first 20 min for warm-up, 30 min for exercise practice, and the last 10 min for dynamic and static stretching, applied to both groups (ERBG, CG). The participants were asked about any history of injury before the study.

### Theraband exercise program characteristics

Theraband Exercises were performed in the ERBG group for 6 weeks (Seguin, Cudlip, Holmes, 2022) (3 days/week) for 45 min after warm-up. Before the application, the ERBG group underwent a one-week adaptation program. After adaptation, the exercise protocol was applied with 1-minute rest between sets in weeks 1–2 (2 sets x 5 repetitions), weeks 2–3 (3 sets x 5 repetitions), and weeks 5–6 (3 sets x 8 repetitions) [38]. (Table 1). The exercises, which included shoulder pull-out, scapular pull-out, lateral raise, front raise, elbow flexion, balance, strength, and stretching movements, were performed with a qualified instructor. A “green-medium resistance” Theraband was chosen because the study group consisted of gymnasts [38]. In the Theraband exercise application, the load of the green bands was standardized according to the percentage of elongation of the band, and the points where they should be held were marked. The resting length of the band was measured and used during the exercise within the 50%–75% elongation range of the band. This elongation level corresponds to an average resistance of 1.45–1.91 kg [39]. The difference value was calculated in the pre/post strength and performance measurements to normalize the increases or decreases in the measured parameters that occurred during resistance band exercises [40]. Rest intervals continued with routine gymnastics training without the Theraband in the CG. The exercise program used in the research was prepared in line with the opinions of expert coaches and academics in the field. The program content was submitted to expert evaluation for its suitability to sports science and training principles, and necessary adjustments were made to finalize it. During this process, the program’s validity and applicability were ensured through expert approval. In addition, sample protocols were used in the preparation of the elastic band exercises [41].

**Table 1:** ERBG exercise application protocol

Theraband Exercise Application Procedure	
1. Open and close the Theraband to the sides. Stand with an upright posture. Extend your arms forward, shoulder-width apart.	
2. Open and close the Theraband to the sides. Stand with an upright posture. Raise your arms shoulder-width apart above your head.	
3. Open and close the Theraband to the sides at chest level. Stand with an upright posture. Rise onto your toes.	
4. Stand upright with your feet shoulder-width apart. Rise onto your toes. Raise your arms shoulder-width apart above your head. Open and close the Theraband to the sides and lower it to your neck.	
5. Pull and release the Theraband with the other arm. Stand upright with your feet shoulder-width apart. Raise one arm. Bend your elbow towards your neck. Grasp one end of the Theraband. Place your other arm behind your back on your waist. Grasp the other end of the Theraband. Keep your forearm stationary.	
6. Open/close the Theraband. Stand with an upright posture. Feet shoulder-width apart. Arms behind hips. Palms facing outwards. Arms close to the torso. Raise arms to hip level.	
7. As you squat down, open your arms to the sides with the Theraband, and close them as you stand up. Stand upright with an upright posture. Bring your right leg forward. Rise onto your toes with the back foot. Place your right foot on the Theraband.	
8. Assume a long sit-up position. Feet facing each other. Feet shoulder-width apart. Place the Theraband between the midsections of your feet. Hold the ends of the band. Lean your body back at a 45-degree angle. Pull and release the Theraband towards your shoulders.	
9. Assume a long sitting position. Feet facing each other. Feet shoulder-width apart. Place the Theraband between the midsections of your feet. Hold the ends of the band. Lean your body back at a 45-degree angle. Open and close the Theraband to the sides.	
10. Lie on your back. Raise your right leg to 90 degrees. Bend your left leg and place it on the floor. Place the Theraband in the middle of your right foot. Keep your arms straight. Keep your head on the floor. Keep your leg straight. Pull and release your leg towards yourself with the Theraband.	
11. Lie face down. Feet shoulder-width apart. Hands at your sides, palms facing upwards. Hold the Theraband at both ends. Open and close your arms. 3 × 5	
12. Lie face down. Feet shoulder-width apart. Arms extended in front. Pull the Theraband towards your chest with your arms.	
13. Place the Theraband upright on the floor. Assume a straight bench position. Your right knee should be on the band. Grasp the end of the band with your right arm. While pulling the Theraband with your right arm, extend your left leg backward.	

### Statistical analysis

Data analysis was performed using SPSS 25.00 (IBM Corp., Armonk, NY, USA) software package with a significance level of *p* < 0.05. Descriptive statistics are presented as mean ± standard deviation, percentage, and frequency. Normality assumption was assessed separately for each variable in the ERBG and CG groups using the Shapiro-Wilk test. Due to the non-normal distribution of the variables (*p* < 0.05) and the small sample sizes of the groups, non-parametric tests were preferred. In this context, the Mann–Whitney U test was used for intergroup comparisons, and the Wilcoxon Signed-Rank test was used for within-group pre-test–post-test comparisons.

## Results

Significant differences were detected in flexibility, balance (left-right foot), muscle power, and back strength (*p* = 0.03) pre- and post-tests in the ERBG group (*p* < 0.05 *p* = 0.01). No differences were observed in body weight, height, BMI, leg strength, hand strength (right-left), and 20 m sprint values (*p* > 0.05; Table 2).

**Table 2:** Comparison of ERBG pretest - posttest motor specifications

Variables	Measurements	x̄	SD	Difference	*p* ^*^
Body Weight (kg)	Pretest	38.28	6.25	0.52	0.64
	Posttest	38.80	5.26		
Height (m)	Pretest	1.45	0.09	0.02	0.09
	Posttest	1.47	0.09		
BMI (kg/m^²^)	Pretest	18.11	1.76	-0.11	0.83
	Posttest	18.00	1.65		
Flexibility	Pretest	44.18	7.88	3,32	0.01 ^**^
	Posttest	47.50	7.15		
Balance (right foot)	Pretest	9.45	4.95	-3.09	0.01 ^**^
	Posttest	6.36	3.91		
Balance (left foot)	Pretest	9.36	5.68	-3,27	0.01 ^**^
	Posttest	6.09	4.09		
Muscle power (cm)	Pretest	23.40	6.23	5.39	0.01 ^**^
	Posttest	28.79	4.57		
Back Strength	Pretest	62.95	20.17	2.15	0.03 ^*^
	Posttest	65.10	19.78		
Leg strength	Pretest	49.73	14.97	3,05	0.12
	Posttest	52.78	12.34		
Hand strength (right)	Pretest	17.19	4.71	0.41	0.65
	Posttest	17.60	5.13		
Hand strength (left)	Pretest	16.44	5.61	0.67	0.52
	Posttest	17.11	5.43		
20 m speed (s)	Pretest	4.3	0.71	-0.35	0.56
	Posttest	3.95	0.44		

*p* ^*^ <0.05

*p* ^**^ <0.01 Difference = (Posttest - Pretest)

In CG, a significant difference was observed only in balance (left foot) values (*p* = 0.01 *p* < 0.05), but no differences were seen in other motor characteristics (*p* > 0.05). No differences were observed in other variables (Table 3).

**Table 3:** Comparison of CG pretest – posttest motor specifications

Variables	Measurement	x̄	SD	Difference	*p* ^*^
Body Weight	Pretest	36.69	10.17	0.91	0.09
	Posttest	37.60	10.69		
Height (m)	Pretest	1.41	0.12	0.02	0.06
	Posttest	1.43	0.11		
BMI (kg/m^²^ )	Pretest	18.17	2.33	-0.06	0.82
	Posttest	18.11	2.88		
Flexibility	Pretest	44.33	4.82	2.37	0.20
	Posttest	46.70	4.04		
Balance (right foot)	Pretest	7.20	5.61	-2.2	0.09
	Posttest	5.00	4.14		
Balance (left foot)	Pretest	7.60	6.13	-1.2	0.01 ^**^
	Posttest	6.40	7.07		
Muscle power	Pretest	21.29	7.05	1.73	0.07
	Posttest	23.02	5.98		
Back Strength	Pretest	59.41	18.92	1,46	0.32
	Posttest	60.87	18.04		
Leg strength	Pretest	50.25	20.14	1.68	0.32
	Posttest	51.93	15.80		
Hand strength (right)	Pretest	19.08	6.98	0.25	0.66
	Posttest	19.33	6.51		
Hand strength (left)	Pretest	17.35	5.58	0.12	0.50
	Posttest	17.47	5.21		
20 m speed	Pretest	4.14	0.82	-0.07	0.42
	Posttest	4.07	0.57		

p^*^ <0.05

*p* ^**^ <0.01 Difference = (Posttest - Pretest)

The study found no significant differences between the CG and ERBG groups in body weight, height, BMI, flexibility, handgrip strength, back strength, leg strength, and 20-meter sprint test measurements (*p* > 0.05). No statistically significant differences were found in motor characteristics between ERBG and CG (*p* > 0.05). Improvements were observed in ERBG and CG in BMI, flexibility, leg balance (foot), muscle power, strength (back, leg, hand), and 20 m speed post-test mean values compared to pre-test mean values. ERBG showed greater improvement in post-test mean values compared to CG, but this improvement was not statistically significant (*p* > 0.05; Table 4).

**Table 4:** Pretest and posttest comparison values of the ERBG and CG

Variables	Measurement	Groups	*N*	x̄	SD	U	*p*
Body Weight (kg)	Pretest	ERBG	11	38.28	6.25	61.00	0.67
		CG	10	36.69	10.17		
	Posttest	ERBG	11	38.80	5.26	61.00	0.74
		CG	10	37.60	10.69		
Height (m)	Pretest	ERBG	11	1.45	0.09	68.50	0.36
		CG	10	1.41	0.12		
	Posttest	ERBG	11	1.47	0.09	68.50	0.36
		CG	10	1.43	0.11		
BMI (kg/m² )	Pretest	ERBG	11	18.11	1.76	58.00	0.94
		CG	10	18.17	2.33		
	Posttest	ERBG	11	18.00	1.65	53.00	0.91
		CG	10	18.11	2.88		
Flexibility	Pretest	ERBG	11	44.18	7.88	59.50	0.96
		CG	10	44.33	4.82		
	Posttest	ERBG	11	47.50	7.15	72.50	0.76
		CG	10	46.70	4.04		
Balance(right foot)	Pretest	ERBG	11	9.45	4.95	83.50	0.34
		CG	10	7.20	5.61		
	Posttest	ERBG	11	6.36	3.91	78.00	0.45
		CG	10	5.00	4.14		
Balance (left foot)	Pretest	ERBG	11	9.36	5.68	73.00	0.50
		CG	10	7.60	6.13		
	Posttest	ERBG	11	6.09	4.09	64.00	0.90
		CG	10	6.40	7.07		
Muscle power (cm)	Pretest	ERBG	11	23.40	6.23	64.00	0.48
		CG	10	21.29	7.05		
	Posttest	ERBG	11	28.79	4.57	86.00	0.06
		CG	10	23.02	5.98		
Back Strength	Pretest	ERBG	11	62.95	20.17	58.00	0.68
		CG	10	59.41	18.92		
	Posttest	ERBG	11	65.10	19.78	61.50	0.62
		CG	10	60.87	18.04		
Leg strength	Pretest	ERBG	11	49.73	14.97	58.00	0.95
		CG	10	50.25	20.14		
	Posttest	ERBG	11	52.78	12.34	61.50	0.89
		CG	10	51.93	15.80		
Hand strength(right)	Pretest	ERBG	11	17.19	4.71	45.50	0.47
		CG	10	19.08	6.98		
	Posttest	ERBG	11	17.60	5.13	56.00	0.50
		CG	10	19.33	6.51		
Hand strength (left)	Pretest	ERBG	11	16.44	5.61	50.00	0.71
		CG	10	17.35	5.58		
	Posttest	ERBG	11	17.11	5.43	49.50	0.88
		CG	10	17.47	5.21		
20 m speed (s)	Pretest	ERBG	11	4.03	0.71	46.50	0.76
		CG	10	4.14	0.82		
	Posttest	ERBG	11	3.95	0.44	53.00	0.57
		CG	10	4.07	0.57		

p^*^<0,05

## Discussion

Elastic band applications were used for centuries in muscle strength training for men, body shaping for women, and developmental motor skill training for children [42], and are actively used in different types of training, such as speed and agility, flexibility, plyometric exercises, and reactive neuromuscular training in current training science literature [43].

The results of the present study showed no significant differences between the CG and ERBG groups in terms of body weight, height, BMI, flexibility, handgrip strength, back strength, leg strength, and 20-meter sprint test measurements. Pre- and Posttests showed *significant changes* in flexibility, balance (left-right foot), muscle power, and back strength values with ERBG exercises and gymnastics training. In the CG group, only balance (left foot) showed a significant difference with continued gymnastics training, but no differences were observed in other motor characteristics.

In the field of gymnastics, Durukan et al. [44] reported significant improvements in motor skills (e.g., single-leg balance, agility, speed, catching, standing long jump, and tennis ball throwing) in 6-year-old children after 16 weeks of 60-minute basic gymnastics training, 3 days a week. Similarly, Paunović et al. [45] reported that developmental gymnastics training improved motor skills more in children compared to physical education classes. At this point, it was found that basic gymnastics training had positive effects on motor characteristics. Aerobic gymnasts also reported that in a 14-week three-step jump test with an elastic band, the band application improved lower extremity and trunk strength significantly [46]. It has also been reported that different forms of application, such as developmental [42] and aerobik gymnastics, contribute positively to the motor development of children [46]. However, it is noteworthy that studies on resistance band (Theraband) applications in the gymnastics branch are limited. This study is expected to fill this gap in the field and contribute to the literature.

Previous studies also reported that non-standard types of resistance training can improve static and dynamic balance abilities in athletes [47]. Gymnasts were reported to have better balance abilities than soccer players, swimmers, and basketball players. The improvement in motor skills resulting from balance training is an increase in the rate of strength development [48]. Proprioceptive training programs effectively improve balance control by optimizing ankle proprioceptive information reweighting, which is beneficial for sports performance and injury prevention. Also, the programs’ reduction in the frequency of ankle sprains in athletes highlights the importance of proprioceptive training in maintaining joint stability and preventing injuries [49]. Gymnastics training was reported to improve proprioception by enhancing joint position awareness and force perception, thereby reducing the risk of injury [50]. The results of our study suggest that the improvement in CG’s left foot balance ability can be attributed to the effects of gymnastics training.

Eccentric exercises performed with Therabands and Theratubes three times a week for four weeks in children were shown to be effective on the quadriceps muscle in terms of strength [25]. The effect of strength training on upper and lower strength and motor performance ability was reported in children aged 7.1–12.3 years, with training once a week or twice a week, showing that strength training programs twice a week support strength development in children [51]. The inclusion of resistance band exercises during water aerobics sessions was recommended to increase upper and lower muscle strength [52]. Positive effects on upper extremity muscle strength and swimming performance have also been reported in child swimmers [53]. Since the study group consisted of participants with long-term gymnastics training, strength training was planned three times a week. This approach demonstrates that the weekly strength training frequency for children is at a sufficient level.

It was also reported that Theraband training, conducted over 9 weeks with 12-year-olds, contributes to the development of shooting performance and some biomotor skills in basketball, and that Theraband training can be effectively applied in terms of strength development during the season [54].

In studies on jumping ability, Therabands were reported to contribute most to the development of spike speed performance, Counter Movement Jump (CMJ) height, and lower extremity peak strength in volleyball players [55]. A study very similar to ours, involving 6 weeks of exercises with elastic resistance bands in 8-9-year-old children, reported that it significantly increased hand grip strength but did not have a significant effect on balance, agility, and flexibility [28]. Flexibility values also showed no significant difference in our study. This is thought to be particularly because of the fact that this age group is in its developmental stage.

Resistance band exercises were reported to improve dribbling, passing, and shooting skills in basketball players [19], coordination and performance in specific drills for soccer players under 15 years of age [22], and vertical jump, horizontal jump, and volleyball performance values in pubertal male volleyball players. It was argued that they should be added as a complementary element in training [20, 55]. Although resistance band applications are common in various sports such as basketball, volleyball, and swimming, their use is limited in gymnastics, particularly in floor gymnastics categories.

It was reported in previous studies that resistance bands used to improve sprint performance showed improvements in 30-meter and 60-meter sprint values, but the best improvement was observed in the 400-meter sprint [56]. Although the study results do not show similarities with our study results, it is considered that this may be because of the 20-meter test in our study, the children age group, and the type of band used.

Previous studies also reported that football players who use resistance band exercises after two different warm-up programs (with and without resistance bands) before training experience improved performance in balance, muscle power, Illinois agility, and 30 m sprint parameters. It is recommended that coaches and athletes regularly incorporate resistance band exercises into training [55], include them in sport-specific warm-up protocols, and apply resistance band exercises before training to achieve high performance in football players [57]. In this context, many researchers recommend adding resistance band exercises to training, but suggest different application times depending on the sport, such as during and after the warm-up process, and during and after training.

Since using elastic resistance bands in training for different populations is versatile and economically feasible [52], and considering its practicality, low cost, and effectiveness in various outcomes, a comparison with traditional devices can clarify and measure the benefits provided by both modes. Meta-analyses of resistance training with elastic devices (tubes and Therabands) and traditional devices (weight machines and dumbbells) on muscle strength have reported no superiority between the analyzed methods for lower or upper extremity muscle strength. Elastic resistance training was reported to provide similar strength gains to traditional resistance training across different population profiles and using various protocols [58].

### Limitations

The study limitations included the limited number of 8-12-year-old girl children who regularly trained as gymnasts, which restricted the study compared to a larger sample size. According to Pinto et al. [52] conducting studies involving interventions with resistance bands is often not possible because of a number of challenges, such as the available space and time for the training program, participant separation, and subject shortages. The research sample consisted of participants from a specific region and a single center, thus limiting the external validity of the findings by restricting their generalizability to different areas and groups. Furthermore, the short duration of ERB applications limits the ability to make a clear interpretation regarding the long-term sustainability of the effects obtained. Although resistance band exercises and gymnastics applications are standard procedures, factors such as trainer experience, motivational approach, and the physical environment in which the applications are performed should be considered when evaluating the findings.

## Conclusions

In conclusion, no significant difference was observed between ERBG and CG. However, ERBG showed an improving effect on flexibility, balance, muscle power, and back strength. The use of ERB exercises in addition to gymnastics had positive effects on motor skills such as flexibility, balance, muscle power, and back strength. We recommend that future research on the use of elastic resistance bands should be prepared considering the application process, application group, application protocol, and the relevant sport.

## Supplementary Information

Supplementary Material 1.

Supplementary Material 2.

## Data Availability

The datasets generated and/or analysed during the current study are not publicly available for privacy reasons but are available from the corresponding author upon reasonable request.

## Declarations

I declare that this study is our own and was conducted in accordance with ethical guidelines.
