# Supplementary material for: The effect of resistance band exercises on motor skills in female gymnasts
Source: BMC Sports Sci Med Rehabil. 2026 Jul 6;18:453. doi: 10.1186/s13102-026-01852-0 (PMC13625460; doi:10.1186/s13102-026-01852-0)
Supplement: Supplementary file 1 [file 13102_2026_1852_MOESM2_ESM.pdf]

T.C.
AFYON KOCATEPE ÜNİVERSİTESİ
SAĞLIK BİLİMLERİ BİLİMSEL ARAŞTIRMA VE YAYIN ETİĞİ KURULU KARARLARI

TOPLANTI SAYISI:09 **KARAR TARİHİ:26.09.2019**

KARAR 2019/28

Üniversitemiz Sağlık Bilimleri Enstitüsü Yüksek Lisans öğrencisi Gül Halime ÇELİK'in "8-12 Yaş Cimnastikcilerde Therabant Egzersizlerinin Motorik Yetiler Üzerine Etkisi" başlıklı yüksek lisans tezi kapsamında kullanacağı veri toplama araçlarının, etik açıdan sakıncalı olmadığına, katılanların oy birliği ile karar verildi.

ASLI GİBİDİR

Prof.Dr.Halil Selçuk BİRİCİK

Sağlık Bilimleri Bilimsel Araştırma ve Yayın Etik Kurulu Başkanı
