# Supplementary material for: The effect of resistance band exercises on motor skills in female gymnasts
Source: BMC Sports Sci Med Rehabil. 2026 Jul 6;18:453. doi: 10.1186/s13102-026-01852-0 (PMC13625460; doi:10.1186/s13102-026-01852-0)
Supplement: Supplementary file 2 [file 13102_2026_1852_MOESM1_ESM.docx]

Editor in Chief

BMC Sports Science, Medicine and Rehabilitation
Dear Editor,

We are pleased to submit our manuscript entitled "The Effect of Resistance Band Exercises on Motor Skills in Female Gymnasts" for consideration for publication in BMC Sports Science, Medicine and Rehabilitation.

This research investigates the effectiveness of elastic resistance bands in improving motor skills in 8-12 year old female gymnasts through routine gymnastics training. The study found that children who used elastic resistance bands alongside gymnastics training showed improved motor skills, but there was no difference compared to the group that only performed routine gymnastics training. Elastic resistance band training has been applied in many sports, but research specifically on gymnastics is extremely limited. It is an economical and useful tool that can be used in gymnastics to prevent injuries, increase motivation in class, and support healthy growth. This study provides a valuable perspective on how elastic resistance band use in gymnastics improves flexibility, balance, leg strength, and vertical jump, contributing to the growing body of evidence regarding its use in both fundamental development and sports performance settings. We believe these findings will be of particular interest to readers interested in rehabilitation, training adaptation, and injury prevention. The manuscript is original, has not been published elsewhere, and is not currently under consideration by any other journal. All authors have read and approved the final version of the manuscript and agree with its submission to BMC Sports Science, Medicine and Rehabilitation.

Sincerely,

Sebiha GÖLÜNÜK BAŞPINAR

Correspondent author
